# Classification of 17 species *Aegilops* using DNA barcoding and SNPs, reveals gene flow among *Aegilops biuncialis*, *Aegilops juvenalis*, and *Aegilops columnaris*

**DOI:** 10.3389/fpls.2022.984825

**Published:** 2022-10-06

**Authors:** Xiaohan Wang, Eunae Yoo, Seungbum Lee, Gyu-Taek Cho, Gi-An Lee, Jung Yoon Yi, Xiaoxuan Du, Seahee Han, Do Yoon Hyun, Nayoung Ro, Kyung-Min Kim

**Affiliations:** ^1^ National Agrobiodiversity Center, National Institute of Agricultural Sciences, Rural Development Administration, Jeonju, South Korea; ^2^ Department of Applied Biosciences, Graduate School, Kyungpook National University, Daegu, South Korea; ^3^ Honam National Institute of Biological Resources, Mokpo, South Korea; ^4^ Korea National University of Agriculture and Fisheries, Jeonju, South Korea

**Keywords:** *Aegilops* spp., genotyping by sequencing, phylogeny, species discrimination, wild wheat

## Abstract

Rapid changes in agricultural environments caused by global warming pose a major challenge to food production and safety. Common wheat (*Triticum aestivum*) is a hexaploid plant (AABBDD) that shares large numbers of quantitative traits and resistance genes with B and D genomes of *Aegilops* species, which are responsible for several metabolic functions and biosynthetic processes, particularly in plant adaptation to biotic as well as abiotic stresses. Comparatively, the abundance of the *Aegilops* gene pool is much higher than that of *Triticum*. Therefore, we used four universal DNA barcodes for plants (ITS2, *mat*K, *rbc*L, and *psb*M*-pet*N) to construct a phylogenetic tree to classify the genus *Aegilops*. Fourteen species were distinguished among a total of 17 representative species. *Aegilops biuncialis*, *Aegilops juvenalis*, and *Aegilops umbellulata* could not be grouped into any of the clusters in the phylogenetic tree, indicating that these three species could not be distinguished by four DNA barcodes. Therefore, from 2408 SNPs obtained using genotyping by sequencing (GBS), we manually screened 30 SNPs that could be potentially used to classify these three species. The results of gene flow and genetic differentiation index (Fst) showed that the genetic differentiation among the three species was small, and there was bidirectional horizontal gene transfer between the three species, which was consistent with our results that the three species were difficult to classify by DNA barcode.

## Introduction

Common wheat (*Triticum aestivum* L.) serves as a source of food for 4.5 billion people worldwide, and grain production of about 730 million tons fulfils 20% of the daily protein requirement (Arzani and Ashraf, 2017). Climate change is anticipated to have adverse effects on the quality and yield of wheat, to counter which, effective strategies for biodiversification of wheat cultivars and promotion of their adaptation to the changing environment are essential. Drastic changes in wheat cultivation environments caused by global warming will pose a major challenge to the environmental adaptability of wheat. During the past 10,000 years, breeders have continuously selected quantitative traits of genetic resources (Nevo et al., 2002), resulting in reduced wheat genetic diversity and depletion of resources with excellent resistance, which has made breeding more difficult (Van Ginkel and Ogbonnaya, 2007). Genetic erosion in wild species and primitive crop plants, and the associated consequences for agriculture, underscore the need to exploit the unrealized potential of ancestral species, many of which have already adapted to harsh environments (Arzani and Ashraf, 2016).

Common wheat is a heterologous hexaploid (AABBDD) with a genome length of approximately 15.07 Gbp (Alonge et al., 2020). *Aegilops* is the genus most closely related to wheat (*Triticum* spp.), and provides important genetic resources for its improvement (Kiani et al., 2021) According to karyotype analysis, *Aegilops* consists of six genomes: U, C, M, N, S, and D (Molnár et al., 2016; Zhao et al., 2018). A diploid wheat (*Triticum monococcum*, AA) was mated with an unknown diploid species (BB) to produce tetraploid durum wheat, which was mated again with a diploid *Aegilops tauschii* (DD) to produce the edible bread wheat *Triticum aestivum* (AABBDD) (He et al., 2019; Pont et al., 2019). The as yet unknown donor species of the B genome is related to the *Sitopsis* section (most likely *Aegilops speltoides* or *Aegilops searsii*), whereas the D genome is provided by *Ae. tauschii*. Many agronomically important traits, including resistance to abiotic and biotic stresses, have been introgressed from *Aegilops* to wheat *via* natural or artificial hybridization (Eastwood et al., 1991; Kolmer, 1996; Ter Steege et al., 2005; Pestsova et al., 2006; Arzani and Ashraf, 2016). The D genome of *Aegilops* is 30 times more diverse than that of *Triticum aestivum* (Fu and Somers, 2009). To broaden the genetic base of cultivated wheat, some breeders employ a hexaploid wheat derived through hybridization of diploid *Aegilops* and tetraploid durum wheat (Valkoun, 2001). Thus, collecting and characterizing the *Aegilops* germplasm is critical to the wheat improvement process.

*Aegilops* is a genus of Eurasian and North American grasses (Tzvelev, 1989; Culumber et al., 2011). Traditionally, *Aegilops* species have been classified based on differences in morphology. However, opinions about the morphological classification of *Aegilops* species differ among taxonomists (Morrison, 1994), and it is difficult for non-taxonomists to accurately classify *Aegilops* using the established criteria (Gupta and Baum, 1989). There are two reasons why it is difficult for non-taxonomists to accurately classify *Aegilops* using established criteria. 1. Instability of the *Aegilops* classification system. Taxonomists have different opinions on the classification criteria of the genus *Aegilops* (Hammer, 1982; Gupta and Baum, 1989; Van Slageren, 1994). 2. Professional terms and the overlapping quantitative range make it difficult to understand and use the taxonomy. Therefore, an easy and reliable method for interspecies classification of *Aegilops* is needed.

DNA barcoding is applied in many fields (Pečnikar and Buzan, 2014), and allows animal, plant, and fungal species to be rapidly identified using one or several standard DNA sequences. Previous DNA barcoding studies of *Aegilops* species mainly sought to identify species that are difficult to distinguish morphologically (Wang et al., 2000; Goryunova et al., 2005; Giraldo et al., 2016; Dizkirici et al., 2016), and phylogenetic analysis of *Aegilops* and *Triticum* species using DNA barcodes (Yamane and Kawahara, 2005; Petersen et al., 2006; Meimberg et al., 2009). However, no studies have applied universal DNA barcodes to identify all species within the *Aegilops* genus. For example, the *Triticeae* tribe was identified using three chloroplast sites, *mat*K, *rbc*L, and *trn*H*-psb*A (Bieniek et al., 2015) and internal transcribed spacer 2 (ITS2) was used to classify 65.51% of Greek *Aegilops* and *Triticum* species (Ganopoulos et al., 2017). The ITS2, *mat*K, *rbc*L, and *trn*H*-psb*A regions are commonly used in plant biometric systems. An additional six chloroplast DNA regions were used to classify *Triticum* species (Awad et al., 2017). The objective of this study was to determine the evolutionary and phylogenetic relationships among *Aegilops* species based on DNA barcoding.

## Materials and methods

### Plant materials, DNA extraction and genotyping by sequencing (GBS)

A total of 17 species (including subspecies) of *Aegilops* (84 accessions) were provided by the Genetic Resource Center of the National Academy of Agricultural Science, Rural Development Administration, Republic of Korea (**Table S1**). To classify these *Aegilops* accessions to the species level using DNA barcoding methods, we selected 2–9 accessions from each species. All samples were taxonomically identified according to the key proposed by Van Salgeren (Van Slageren, 1994). Leaf tissues were harvested during the tillering stage. Total DNA was extracted using the DNeasy Plant Mini kit (Qiagen, Valencia, CA, USA) according to the manufacturer’s instructions. The DNA was dissolved in 100 μL of water and diluted to 20 ng/μL. Genomic DNA was quantified using a Nanodrop/UVS-99 instrument (ACTGene, Kendall Park, NJ, USA), and the A260/A280 ratio was calculated. DNA quality was verified on a 1% agarose gel, and the DNA was stored at −20°C.

Genotyping by sequencing (GBS) was performed for the 14 accessions of three species (*Aegilops biuncialis*, *Aegilops juvenalis*, *Aegilops columnaris*). The GBS libraries were sequenced in the Illumina NextSeq500 (Illumina, San Diego, CA, USA) with the length of 150 bp single-end reads. Genome analysis toolkit 3.7 (GATK) was used to perform local realignments of reads to correct misalignments due to the presence of indels (“RealignerTargetCreator” and “IndelRealigner” arguments). The “HaplotypeCaller” and “SelectVariants” arguments were used for calling candidate SNPs aligned to *Aegilops*_*tauschii*.Aet_v4.0 reference genome. Filtering was performed using Tassel 5 (filter genotype table sites: site max allele frequency = 0.95, max missing data = 0.05, max heterogeneous proportion = 0.2), on 6338 SNPs to yield 2408 SNPs. Imputation was not performed.

### Polymerase chain reaction amplification and sequencing

The ITS2 nuclear region and nine chloroplast regions were used to analyze the 84 accessions from 17 *Aegilops* species, and to evaluate the applicability of the 10 DNA barcode regions. The sequence of universal primers and PCR conditions for the DNA barcode regions were adopted from previous studies (Chen et al., 2010; Awad et al., 2017; **Table S1**). The total volume of the PCR reaction was 20 μL, and contained 1× PCR buffer, 0.1 mM primer, 0.2 mM dNTP, 1 U Taq DNA polymerase, and 200 ng of template DNA. The universal primer set was used for all reactions, as follows: 95°C for 5 min, followed by 30 cycles at 95°C for 20 s, all 10 pairs primers used an annealing temperature of 55°C for 40 s, and 72°C for 30 s, and a final extension at 72°C for 10 min. The temperature was then maintained at 15°C, and the PCR products were visualized on 1.5% agarose gels. PCR product sequencing was performed by Macrogen (Seoul, South Korea). Forward and reverse sequences were assembled and aligned for consensus using BioEdit v7.2.5 software. Sequence data was uploaded to GenBank (https://www.ncbi.nlm.nih.gov/genbank/), and GenBank ID is shown in **Table S1**.

### Data analysis and phylogenetic analysis based on barcoding regions

The consensus sequence for each region was manually edited using MEGAX software (Kumar et al., 2018) and aligned using the ClustalW program. Manual adjustments were made to the nucleotide sequence of the barcode region to improve alignment. All variable sites were re-checked against the original trace file. To assess barcode gaps, the distribution of intraspecific and interspecies distance was calculated using the TAXON DNA program (Meier et al., 2006) based on a pairwise distance model corrected using Kimura-2 parameters (K2P). The result retains four decimal places. Intraspecific/interspecies distance ratio (Three significant figures are retained) = Mean intraspecific distance (the raw data before rounding)/Mean interspecies distance (the raw data before rounding). MEGAX was used to construct a neighbor-joining (NJ) tree through a K2P model. For each DNA barcode region, the best-fit replacement model was tested using IQ-TREE v1.6.2 software (Nguyen et al., 2015). According to the test results, we constructed maximum likelihood trees using the best-fit replacement model of the candidate DNA barcodes (**Table S2**). Figtree v1.4.4 software (Rambaut, 2006) was used for visualization. Species discrimination was considered successful only when all individuals of the same species formed a single clade. Species identification was assessed using bootstrap values (Liu and Singh, 1992). We tested the individual-level discrimination rate of each marker based on the best match, best close match, and bio-barcode amplification (BBA) results of all species; based on the TAXONDNA results; all possible combinations were used in the K2P-corrected distance model (Meier et al., 2006). Nucleotide diversity and Tajima’s D, which reflects the selection pressure experienced by the candidate region, were calculated using the DnaSP v5 program (Librado and Rozas, 2009).

### Migration and genetic differentiation index (Fst)

Migrate-n v5.0.4 software (Beerli and Palczewski, 2010) was used to estimate gene flow of 3 species (*Ae. biuncialis*, *Ae. juvenalis*, *Ae. columnaris*). The data was entered into a SNP data model, the sampling increment was set to 100000, and the number of steps in the chain was set to 10000. We used a Brownian motion model to calculate theta values and effective mobility in two directions, and Bayesian analysis to calculate posterior distribution. Model 2 (Beerli et al., 2019) was used to analyze gene flow between the three species. The pairwise Fst among the three species was analyzed using GenAlex v6.5 (Peakall and Smouse, 2006).

## Results

### Evaluation of intra and inter species variation of candidate barcodesintraspecific

**Figure 1:**
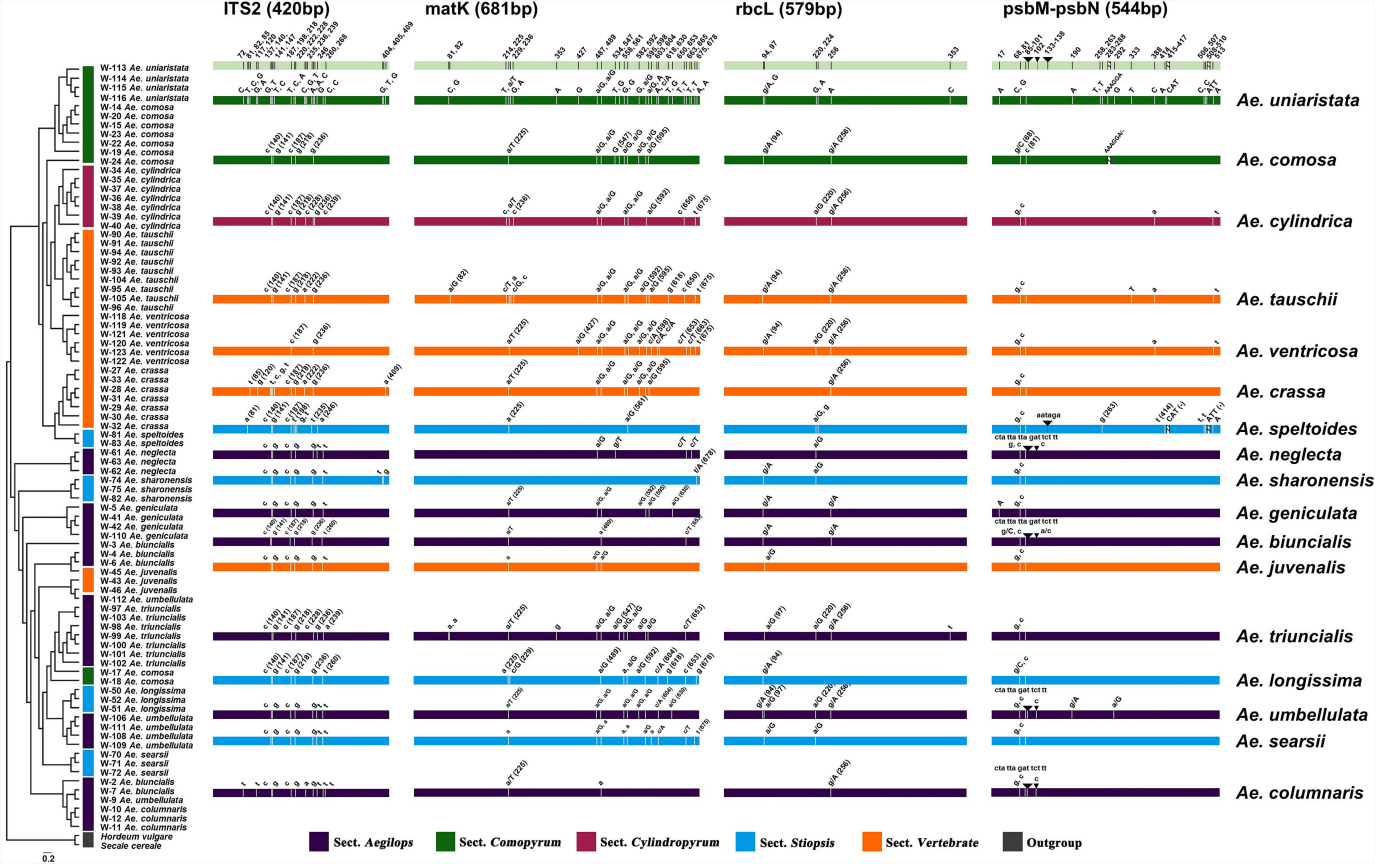
Differential distribution of four DNA barcode regions among 17 *Aegilops* species. Sections of species represented by different colors are described in the bar below. Uppercase and lowercase letters indicate the same and different nucleotides as the reference gene (*Ae. uniaristata*), respectively. The numbers in parentheses indicate the position of the DNA barcode region.

The PCR and sequencing success rates for both regions were 100%. There was one insertion site in ITS2, with a sequence length of 420 bp (**Figure 1**; **Table 1**). There were no insertion/deletion sites in *mat*K, which had a sequence length of 681 bp and the highest nucleotide diversity (0.00863). The ITS2 region had 25 variant sites, with low nucleotide diversity (0.00035); the *mat*K region had 28 variant sites, with a nucleotide diversity of 0.00863; and *psb*M*-pet*N had 6 variant sites. A Tajima’s D < 0 for ITS2 and *psb*M*-pet*N indicated that the rare allele was present at high frequency. A Tajima’s D > 0 for *mat*K and *rbc*L indicated that the rare allele was present at low frequency.

**Table 1:** Universal primer sequences and polymerase chain reaction conditions for 10 DNA barcode regions.

DNA barcode	Forward primer	Reverse primer	Reference sequence length (bp)
*psb*M*-pet*N	ATTCCAATTAATCATTGAAG	TACTACTAATTGATTAAGTAA	624
*trn*G*-trn*T	ATAAAAAGTTTAGTC/aTAGTT	TTTGTCCACCAGTTTCTGGTAC	529
*psa*A*-ycf3*	CCGCCAACTGTCTTTTTAGT	TTGGTTGTTGATCCATTAATC	610
*atp*B*-rbc*L	GACCCAAATTGTCAACAGGC	AGGCACAGATCCTCCACAAAAGGCA	630
*pet*A*-psb*J	AATTGCTAGAATTATCTATG	TGAAAAAGTAGGAGCTTAGCG	740
*rpl32-trn*L	GTGTCGAATTACTCGGTACA	CTTCAAATAATAGGTAACTTAAAAG	650
ITS2	ATGCGATACTTGGTGTGAAT	GACGCTTCTCCAGACTACAAT	427
*mat*K	CGTACAGTACTTTTGTGTTTACGAG	ACCCAGTCCATCTGGAAATCTTGGTTC	711
*psb*A*-trn*H	GTTATGCATGAACGTAATGCTC	CGCGCATGGTGGATTCACAATCC	602
*rbc*L	ATGTCACCACAAACAGAGACTAAAGC	GAAACGGTCTCTCCAACGCAT	595

**Figure 2:**
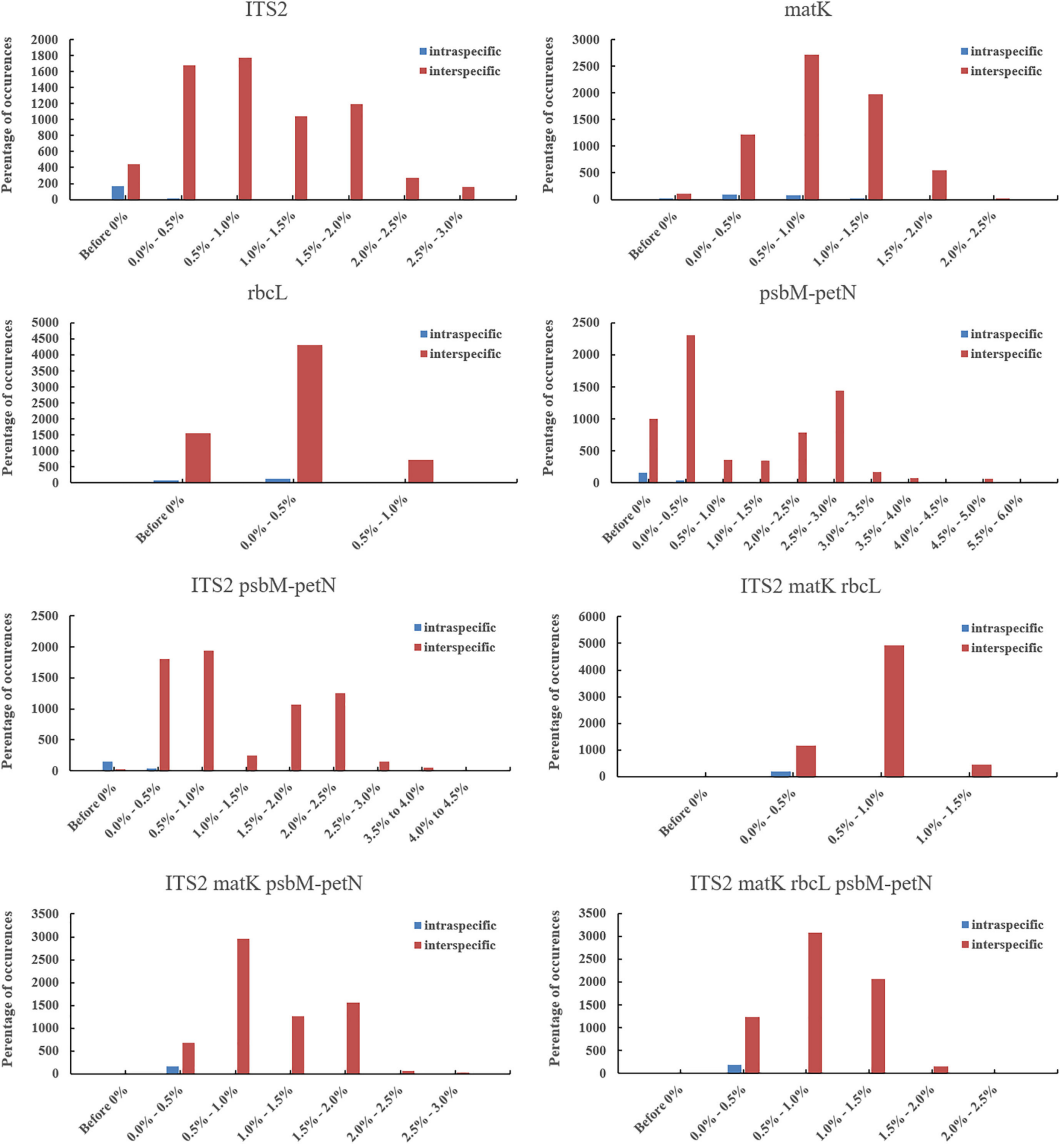
Interspecies and intraspecific distance distributions of candidate DNA barcodes. Blue represents the distance between intraspecific accessions, and red represents distance between pairwise different species accessions.

To create a barcoding gap, the interspecies distance of the barcode must be significantly larger than the intraspecific distance. The ITS2, *mat*K, *rbc*L, *psb*M-*pet*N barcode regions provided the most interspecies variation. The interspecies distance range of all the four candidate DNA barcodes (including combinations) was 0–5.67% (**Figure 2**) and primarily distributed in the range 0–3.0%. Only *psb*M*-pet*N had a non-normal interspecies distance distribution, and only *psb*M*-pet*N had an interspecies distance > 4.5%. The range of intraspecific distance was 0–1.26%. The average intraspecific distance of *mat*K was 0.00489. The range of the intraspecific/interspecies distance ratio among four candidate DNA barcodes and their combinations was 0.009–0.741. The ITS2 region had the lowest intraspecific/interspecies distance ratio (0.009). The intraspecific/interspecies distance ratio of *rbc*L was the highest, at 0.741. The intraspecific and interspecies distance distribution intervals for each candidate barcode sometimes overlapped, but this phenomenon was rarest for ITS2. In addition, we listed the number of intraspecific variation characters for each species in ITS2, *mat*K, *rbc*L, *psb*M-*pet*N barcode regions (**Table S3**). The results showed that ITS2 provided the lowest intraspecific resolution, with only one intraspecific variation identified in *Aegilops comosa*. The *mat*K region provides the highest intraspecific variation. Both *rbc*L and *mat*K had variation within 17 species. The *psb*M-*pet*N region only provides intraspecific variation of *Aegilops biuncialis*, *Aegilops comosa*, *Aegilops longissima*, *Aegilops speltoides*, *Aegilops tauschii*, and *Aegilops umbellulata*.

### Candidate barcode discrimination rate analysis

The BBA test was used to analyze the discrimination ability of the candidate barcodes (**Table S4**). In the best match analysis, the queries identified the closest barcode matches. The identification was considered correct when both sequences were from the same species. Any mismatch was considered incorrect. Several cases with equally good best matches from different species were considered ambiguous (Raveendar et al., 2017). In the best close match analysis, all queries that did not have a barcode match below the threshold were not recognized, and were compared to the species identity of the most recent barcode. If the names were identical, the query was considered correct. For the single barcode, best match, and best close match tests, the discrimination rate of *rbcL* was low (5.81%); the identification rate of ITS2 was higher (56.97%). When I, M, R and P were combined, the discrimination rate was 88.37%.

### Phylogenetic analysis

**Figure 3:**
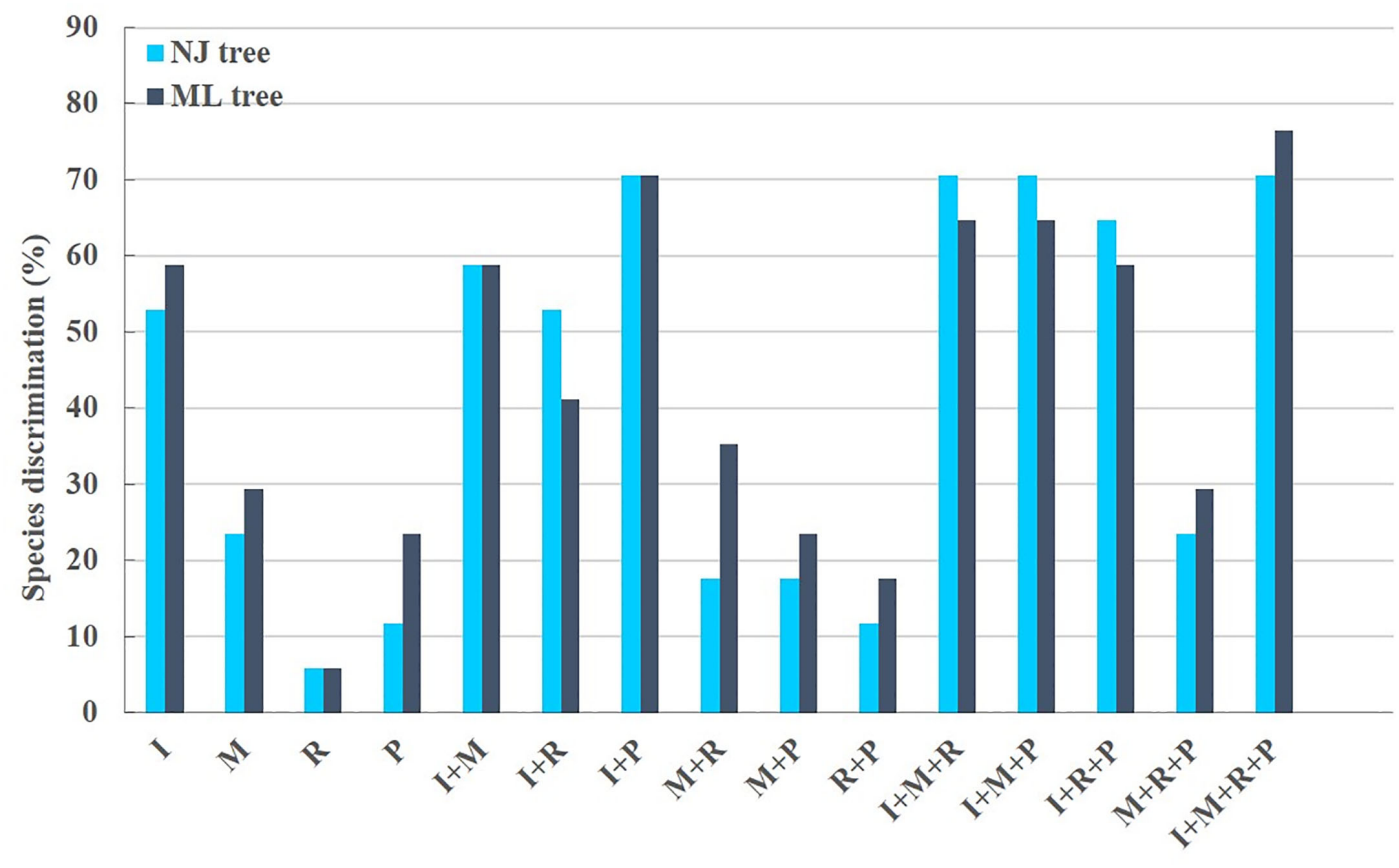
*Aegilops* species discrimination rates of maximum likelihood (ML) tree and neighbor-joining (NJ) tree in each candidate DNA barcode region. R, *rbc*L; M, *mat*K; P, *psb*M-*pet*N; and I, ITS2. Light blue and dark blue represent the *Aegilops* species discrimination rate of NJ tree and ML tree, respectively.

The phylogenetic results showed that none of the 15 candidate barcodes or barcode combinations could classify all species. However, in all phylogenetic trees, *Aegilops* formed a monophyletic clade with high bootstrap support (100%). Among the four single barcodes, ITS2 had the highest discrimination ability (NJ tree: 52.94%, ML tree: 58.82%) (**Figure 3**). As shown in **Figure 3**, the ML tree as a whole exhibits a higher barcode discrimination ability than the NJ tree, DNA barcode combinations have higher discriminative ability than single DNA barcodes. The discrimination ability of *psbM-petN* was lower than those of ITS2 and *mat*K (NJ tree: 11.76%, ML tree: 23.53%); however, it classified *Aegilops geniculate*, which other barcodes could not distinguish. Finally, *rbcL* had the lowest discrimination ability (NJ and ML trees: 5.88%). Among the 11 barcode combinations, I + M + R + P had the highest discrimination ability (NJ tree: 70.59%, ML tree: 76.47%). We list the identification effects of ITS2, *mat*K, *rbc*L, *psb*M-*pet*N and their combinations in the maximum likelihood (ML) tree (**Table S5**). Ten species *Ae. searsii*, *Ae. comosa*, *Ae. crassa*, *Ae. tauschii*, *Ae. speltoides*, *Ae. uniaristata*, *Ae. ventricosa*, *Ae. columnaris*, *Ae. neglecta*, *Ae. sharonensis* can be distinguished by ITS2. *Aegilops cylindrica*, *Ae. geniculata*, *Aegilops triuncialis*, *Aegilops longissima* need to be differentiated by I + M + R + P. *Ae. comosa* could only be distinguished by a single ITS2, but not by the I+M+R+P combination. Among the 17 *Aegilops* species, 14 were successfully identified using the present method; whereas, *Aegilops biuncialis*, *Aegilops juvenalis*, and *Aegilops umbellulata* were observed to fall into an intermediate taxonomic level between genus and species. Next, we attempted to organize *Aegilops* into sections based on existing DNA barcodes; the results for 15 barcodes (including their combinations) are shown in (**Figure S1**). None of the barcodes was able to aggregate the accessions into sections, except for Sect. *Cylindropyrum*. We found that *Ae. uniaristata* and *Ae. comosa* belong to Sect. *Comopyrum* and are distributed in the ITS2 region, whereas other clusters were distributed in the I + M + R + P region. Similarly, part of Sect. *Aegilops* and part of Sect. *Stiopsis* were distributed deep within the ITS2 region, but were also separately clustered in the I + M + R + P region.

### Gene flow and differentiation index among *Ae. biuncialis*, *Ae. juvenalis*, and *Ae. columnaris*

We believe that gene flow may exist between the three species that cannot be distinguished by DNA barcodes, and the genetic differentiation index is low. Population size (Theta) and migration rate (M) were calculated using Migrate-n Version 5.0.4. The results showed that *Ae. biuncialis*, *Ae. juvenalis*, and *Ae. columnaris* had similar bidirectional migration rates, and the symmetric bidirectional migration model could explain the data (**Table 2**).

**Table 2:** Migration rates (average of two independent runs) between tree species (*Ae. biuncialis*, *Ae. juvenalis*, *Ae. columnaris*).

Migration direction		Migrate rate	Migration direction		Migrate rate
From	To	(Mean)	From	To	(Mean)
*Ae. biuncialis*	*Ae. juvenalis*	449.013	*Ae. juvenalis*	*Ae. biuncialis*	475.882
*Ae. biuncialis*	*Ae. columnaris*	522.129	*Ae. columnaris*	*Ae. biuncialis*	496.552
*Ae. juvenalis*	*Ae. columnaris*	449.733	*Ae. columnaris*	*Ae. juvenalis*	507.885

Pairwise Fst was analyzed using 6338 SNPs for three species (*Ae. biuncialis*, *Ae. juvenalis*, *Ae. columnaris*). The Fst between *Ae. biuncialis* and *Ae. juvenalis*, *Ae. biuncialis* and *Ae. columnaris*, *Ae. juvenalis* and *Ae. columnaris* were 0.129, 0.106 and 0144, respectively. The genetic differentiation index among the three species was between 0.05 and 0.15, and the degree of differentiation was small.

### Classification based on genotyping by sequencing (GBS) data

In the genotyping by sequencing (GBS) results of three species (*Ae. biuncialis*, *Ae. juvenalis*, *Ae. columnaris*), SNPs that can be used to distinguish species were screened. The aim is to provide a complete molecular classification key, combined with DNA barcodes to classify the 17 species in the genus *Aegilops*. Among the 2408 SNPs obtained after quality control, a total of 30 markers that could be used potentially for differentiating the three species (*Ae. biuncialis*, *Ae. juvenalis*, *Ae. columnaris*) were selected (**Table 3**).

**Table 3:** Information on 30 SNPs used to classify *Ae. biuncialis*, *Ae. juvenalis*, and *Ae. columnaris* and their polymorphisms in three species.

No.	Major allele frequency	Alleles	chromosome	Position	*Ae. biuncialis*					*Ae. juvenalis*			*Ae. columnaris*					
					W-2	W-3	W-4	W-6	W-7	W-43	W-45	W-46	W-9	W-106	W-108	W-109	W-111	W-112
1	0.78571	C/G	1D	170914803	C	C	C	C	C	G	G	G	C	C	C	C	C	C
2	0.78571	A/G	1D	170914885	A	A	A	A	A	G	G	G	A	A	A	A	A	A
3	0.57143	G/C	1D	402694752	G	G	G	G	G	G	G	G	C	C	C	C	C	C
4	0.71429	C/G/A	1D	438741637	C	C	C	C	C	G	G	G	A	C	C	C	C	C
5	0.57143	G/A	1D	446643840	G	G	G	G	G	G	G	G	A	A	A	A	A	A
6	0.57143	T/G/A	2D	154402884	G	G	G	G	G	T	T	T	T	T	T	T	A	T
7	0.64286	C/A	2D	219835561	A	A	A	A	A	C	C	C	C	C	C	C	C	C
8	0.78571	C/G	2D	323454908	C	C	C	C	C	G	G	G	C	C	C	C	C	C
9	0.57143	G/A	2D	376884273	G	G	G	G	G	G	G	G	A	A	A	A	A	A
10	0.64286	T/G	2D	398965931	G	G	G	G	G	T	T	T	T	T	T	T	T	T
11	0.64286	C/T	2D	398966000	T	T	T	T	T	C	C	C	C	C	C	C	C	C
12	0.71429	C/T/A	3D	15908948	C	C	C	C	C	T	T	T	A	C	C	C	C	C
13	0.78571	T/C	3D	50158698	T	T	T	T	T	C	C	C	T	T	T	T	T	T
14	0.57143	G/A	3D	91598837	G	G	G	G	G	G	G	G	A	A	A	A	A	A
15	0.57143	C/T	3D	110978366	C	C	C	C	C	C	C	C	T	T	T	T	T	T
16	0.57143	G/A	3D	123666416	G	G	G	G	G	G	G	G	A	A	A	A	A	A
17	0.57143	A/G	3D	173185540	A	A	A	A	A	A	A	A	G	G	G	G	G	G
18	0.57143	C/T	3D	211653623	C	C	C	C	C	C	C	C	T	T	T	T	T	T
19	0.78571	G/T	3D	243220047	G	G	G	G	G	T	T	T	G	G	G	G	G	G
20	0.57143	A/T	3D	325280760	A	A	A	A	A	A	A	A	T	T	T	T	T	T
21	0.57143	G/C	4D	98966982	G	G	G	G	G	G	G	G	C	C	C	C	C	C
22	0.57143	G/T	4D	106342034	G	G	G	G	G	G	G	G	T	T	T	T	T	T
23	0.57143	A/G	4D	106343975	A	A	A	A	A	A	A	A	G	G	G	G	G	G
24	0.78571	C/G	4D	359828556	C	C	C	C	C	G	G	G	C	C	C	C	C	C
25	0.57143	A/G	4D	411973293	A	A	A	A	A	A	A	A	G	G	G	G	G	G
26	0.78571	A/C	5D	95729416	A	A	A	A	A	C	C	C	A	A	A	A	A	A
27	0.78571	C/G	5D	95729417	C	C	C	C	C	G	G	G	C	C	C	C	C	C
28	0.78571	G/C	5D	283515563	G	G	G	G	G	C	C	C	G	G	G	G	G	G
29	0.57143	A/G	6D	404358451	A	A	A	A	A	A	A	A	G	G	G	G	G	G
30	0.78571	G/A	7D	198673099	G	G	G	G	G	A	A	A	G	G	G	G	G	G

## Discussion

In the present study, we selected ITS2 as the ITS candidate barcode. Although only the second intron was used in this study, 26 variant sites showed a nucleotide diversity (π) of 0.001 (**Table 4**). Our sequence alignment and phylogenetic analysis results showed that ITS2 was able to identify 10 *Aegilops* species. The intraspecific/interspecies distance ratio was 0.105, with a BBA correct match rate of 56.97%, indicating high accuracy. The ITS2 region is widely used in animal, plants, algae, fungi and prokaryotes identification and phylogenetic studies (Rubinoff et al., 2006), due to its multiple copy numbers, small size, and other useful characteristics, which allow easy amplification. The ITS2 region is highly variable even between closely related species (Song et al., 2012) due to the relatively low evolutionary pressure on this barcode, which was confirmed in this study by a Tajima’s D that was significantly lower than 0 (**Table 4**). The ITS region is among the best candidate barcodes for plants. Although ITS1 has high nucleotide diversity, it has poor universality (Han et al., 2013). Previous studies have applied ITS1 to successfully distinguish *Ae. caudata*, *Ae. tauschii*, *Ae. uniaristata*, *Ae. speltoides*, *Ae. cylindrica*, *Ae. triuncialis*, and *Ae. neglecta* (Goryunova et al., 2005; Dizkirici et al., 2016). In a previous study, 10 *Aegilops* species were distinguished based on the ITS (Dizkirici et al., 2016), including *Ae. umbellulata* and *Ae. biuncialis*; these were not classified in the present study, likely because we read only the second ITS intron. In a future study, we will read the first ITS intron and merge the data to classify these two species.

**Table 4:** Information and genetic diversity of candidate DNA barcode regions.

DNA barcode	Aligned Length (bp)	Variable characters	Nucleotide diversity(π)	Tajima’s test (D)	Mean intraspecific distance	Mean interspecies distance	Intraspecific/interspecies distance ratio
I	437	26	0.001	−0.882	0.0010	0.0095	0.1048
M	712	28	0.009	0.175	0.0049	0.0091	0.5374
R	582	6	0.002	0.150	0.0017	0.0022	0.7399
P	688	10	0.003	−0.672	0.0010	0.0126	0.0767
I + M	1149	54	0.009	−0.356	0.0031	0.0093	0.3301
I + R	1019	19	0.005	−0.700	0.0010	0.0054	0.1828
I + P	1125	36	0.005	−0.883	0.0006	0.0114	0.0551
M + R	1294	34	0.006	0.182	0.0034	0.0060	0.5691
M + P	1400	38	0.006	−0.075	0.0030	0.0108	0.2745
R + P	1270	16	0.002	−0.389	0.0013	0.0079	0.1626
I + M + R	1731	60	0.007	−0.303	0.0026	0.0069	0.3754
I + M + P	1837	64	0.007	−0.433	0.0023	0.0105	0.2165
I + R + P	1707	42	0.004	−0.740	0.0010	0.0083	0.1181
M + R + P	1982	44	0.005	−0.038	0.0026	0.0083	0.3096
I + M + R + P	2419	70	0.006	−0.381	0.0021	0.0085	0.2497

I, ITS2; M, matK; R, rbcL; P, psbM-petN.

The *mat*K gene is among the most variable angiosperm coding genes, and has been considered a barcode for terrestrial plants (Yu et al., 2011). However, *mat*K performed poorly in our phylogenetic analysis. The extensive divergence in the *mat*K sequence among higher taxa can result in questionable relationships within some phylogenetic clades (Yu et al., 2011), which is consistent with our results. Nonetheless, although it did not perform as well as ITS2, *mat*K successfully identified five species not identified by ITS2. The *mat*K gene also classified *Ae. searsii*.

The *mat*K gene has been used to classify the *Aegilops* species *Ae. cylindrica*, *Ae. tauschii*, *Ae. sharonensis*, and *Ae. longissima* (Dizkirici et al., 2016). We studied three accessions of *Ae. sharonensis*; in the *mat*K region, multiple mutation sites resulted in greater intraspecific than interspecies distance for *Ae. sharonensis*, such that we were unable to classify this species. Our result differs from that of Dizkirici et al. (2016), who used only one *Ae. sharonensis* accession.

The chloroplast *psb*M*-pet*N gene is less able to discriminate *Triticum* species (Awad et al., 2017). However, while pre-selecting candidate barcodes, we found that *psb*M*-pet*N had the ability to discriminate *Aegilops* species. Although *psb*M*-pet*N classified only three species, it yielded the highest intraspecific/interspecies distance ratio (0.077) because the 21-bp indel appeared at 98–119 bp. *Aegilops umbellulata*, *Ae. biuncialis*, *Ae. columnaris*, and *Ae. neglecta* formed a cluster that exhibited significant differences from other species. The largest contribution of *psb*M*-pet*N to this study was the classification of *Ae. geniculata*, *Ae. biuncialis*, *Ae. juvenalis*, and *Ae. umbellulata*, which are difficult to distinguish using other candidate barcodes.

In this study, we selected the most commonly used barcodes (ITS2, *mat*K, *rbc*L, and *pasA-trnH*) for delimitation of plant species. We also analyzed chloroplast sequences of each *Aegilops* species obtained from GenBank, to identify the most diverse barcode regions, and determined that six regions had high nucleotide diversity. We sequenced these six candidate DNA barcodes and constructed a phylogenetic tree. The results indicated that *psbM-petN* could distinguish *Ae. geniculata*, which cannot be identified by other DNA barcodes. However, none of the other five candidate DNA barcodes effectively distinguished *Aegilops* species.

In this study, the most effective DNA barcode for distinguishing members of genus *Aegilops* was the combination of I + M + R + P. Using tree-based and BBA methods to compare the results obtained from 15 candidate barcodes, we found that the I + M + R + P combination had the highest identification ability (**Table S4**). In the best match analysis of I + M + R + P, the rate of correct identifications was 88.37%. This combination distinguished 13 species, including *Ae. uniaristata*, *Ae. cylindrica*, *Ae. tauschii*, *Ae. ventricosa*, *Ae. crassa*, *Ae. speltoides*, *Ae. neglecta*, *Ae. sharonensis*, *Ae. geniculata*, *Ae. triuncialis*, *Ae. longissima*, *Ae. searsii*, and *Ae. columnaris*. *Aegilops comosa* was distinguished by ITS2. Among the 17 *Aegilops* species, 14 were distinguished. The three species not identified using our method were *Ae. biuncialis*, *Ae. juvenalis*, and *Ae. umbellulata*. However, the barcode combination increases the difficulty of the analysis compared to a single locus. The inability of DNA barcodes to classify all species in genus *Aegilops* is due not only to a lack of variation, but also to differences between the genetic trees of the plastid genes and species boundaries. Thus, the application of combined loci does not eliminate the inherent deficiencies of plant DNA barcoding.

To determine why ITS2 performed better than the chloroplast region barcode in terms of species differentiation, we compared the site information of ten candidate barcodes. The ITS2 evolution rate is higher than that of the chloroplast region barcode because ITS2 is located in the rDNA region of the nuclear genome, and is susceptible to recombination. The chloroplast genome of *Triticeae* species is maternally inherited, in a manner similar to the cytoplasmic genome of most angiosperms (Luo et al., 2012). Therefore, although ITS2 is more highly conserved than chloroplast genes, ITS2 has a faster evolution rate; thus, most ITS2 variable sites are single-nucleotide polymorphisms with interspecies specificity. The *psb*M*-pet*N variant site with higher nucleotide diversity contains many larger fragments (5–20 bp). The main reasons for these mutation sites are base transversion, repetitions of tandem repeats, and indels. Most of the variation is not unique, but instead shared by several species, indicating that the evolution rates of chloroplast candidate barcodes are relatively slow. Therefore, this variation shared across species is not sufficient to provide interspecies discrimination of *Aegilops.* However, *Aegilops* species can be clearly classified using additional barcodes to complement the classification results of ITS2.

In the current study, *Aegilops biuncialis*, *Ae. juvenalis*, and *Ae. umbellulata* were not identified when barcodes were used. The results of gene flow and Fst showed that the genetic differentiation among the three species was small, and there was bidirectional horizontal gene transfer between the three species. *Ae. biuncialis*, *Ae. juvenalis*, and *Ae. columnaris* are distributed in Iraq, Syria, and Azerbaijan, and have sufficient geographical conditions for gene flow (Germplasm Resources Information Network, 2022. http://npgsweb.arsgrin.gov/gringlobal/taxon/taxonomydetail?

id=100015, Accessed September 7 2022). Previous studies have analyzed C-banding patterns and found that all species exhibit extensive C-banding polymorphisms and a high frequency of chromosomal rearrangements. *Aegilops biuncialis* is the result of crossing *Ae. umbellulata* with other diploid species of the same genus (Badaeva, 2002), which makes it more difficult to identify using DNA barcodes, which was consistent with our results. We provided 30 SNPs to distinguish the three species. The limitations of DNA barcoding in taxonomic and phylogenetic analysis are increasingly shown. With the rapid development of sequencing technology, phylogenetic analysis using whole genome sequences (wider coverage) can effectively avoid these errors.

## Data availability statement

The datasets presented in this study can be found in online repositories. The names of the repository/repositories and accession number(s) can be found below: https://www.ncbi.nlm.nih.gov/genbank /, MW447521.

## Author contributions

G-TC, G-AL, EY, NR, DH, and K-MK conceived and designed the experiments. XW and SH performed the experiments and analyzed the data. XW and DH project coordination and analysis. XW contributed materials. XW, G-AL, JY, SH, XD, SL, and EY drafted the manuscript and figures. All authors contributed to the article and approved the submitted version.

## Funding

This project was supported by the Research Program for Agricultural Science & Technology Development (Project No. PJ01491905).

## Conflict of interest

The authors declare that the research was conducted in the absence of any commercial or financial relationships that could be construed as a potential conflict of interest.

## Publisher’s note

All claims expressed in this article are solely those of the authors and do not necessarily represent those of their affiliated organizations, or those of the publisher, the editors and the reviewers. Any product that may be evaluated in this article, or claim that may be made by its manufacturer, is not guaranteed or endorsed by the publisher.
