# Supplementary material for: Classification of 17 species Aegilops using DNA barcoding and SNPs, reveals gene flow among Aegilops biuncialis, Aegilops juvenalis, and Aegilops columnaris
Source: Front Plant Sci. 2022 Oct 6;13:984825. doi: 10.3389/fpls.2022.984825 (PMC9583012; doi:10.3389/fpls.2022.984825)
Supplement: Supplementary file 1 [file Table_1.pdf]

Supplementary

1 Supplementary Figures and Tables

1.1 Supplementary Figures

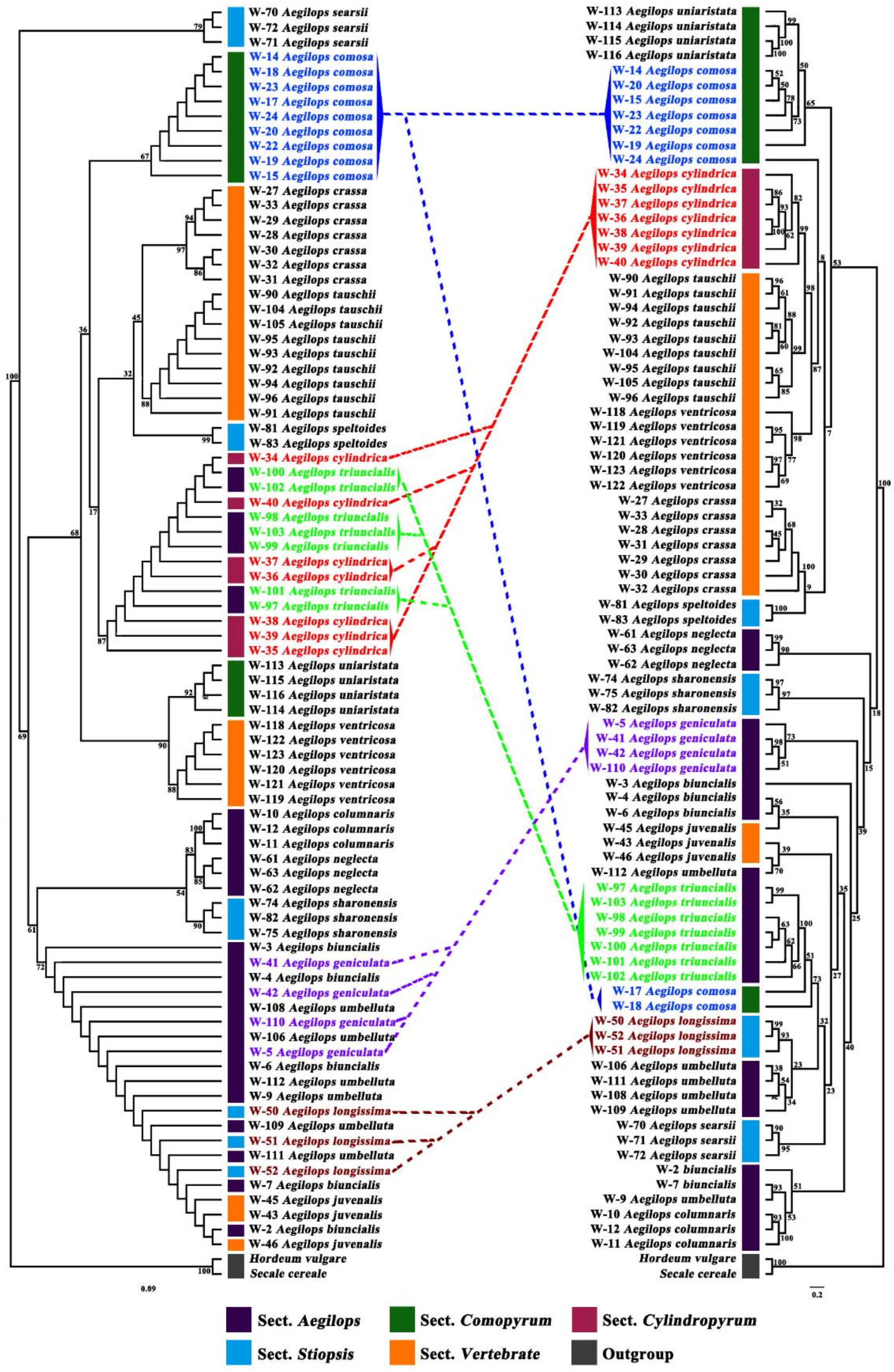

Supplementary Figure 1. (Left) ITS2 and (right) combined ITS (I) + *mat*K (M) + *rbc*L (R) + *psb*M-*pet*N (P) maximum likelihood trees for *Aegilops*. Posterior probability values are shown above the branches. Colored bars indicate sections.

1.2 Supplementary Tables

Supplementary Tables 1. Detailed descriptions of 84 *Aegilops* accessions. (1/4)

No.	Sample No.	Taxon	Geographical distribution*	Acc. No.	Section	Origin	ITS2	*mat*K	*rbc*L	*psb*M-*pet*N
1	W-2	*Ae. biuncialis*	DZA, LBY, MAR, TUN, CYP, IRN, IRQ, ISR, JOR, LBN, SYR, TUR, RUS, ARM, AZE, GEO, RUS, UKR, ALB, BGR, GRC, HRV, ITA, MKD, ROU, SRB, ESP, FRA	K024016	*Aegilops*	-	MW447521	OM314822	OM222954	OL417401
2	W-3	*Ae. biuncialis*		801590	*Aegilops*	RUS	MW447522	OM314823	OM222955	OL417402
3	W-4	*Ae. biuncialis*		801589	*Aegilops*	RUS	MW447523	OM314824	OM222956	OL417403
4	W-6	*Ae. biuncialis*		208978	*Aegilops*	UKR	MW447524	OM314825	OM222957	OL417404
5	W-7	*Ae. biuncialis*		204618	*Aegilops*	-	MW447525	OM314826	OM222958	OL417405
6	W-10	*Ae. columnaris*	IRN, IRQ, LBN, SYR, TUR, ARM, AZE, GRC	208980	*Aegilops*	ARM	MW447526	OM314827	OM222959	OL417406
7	W-11	*Ae. columnaris*		189841	*Aegilops*	ARM	MW447527	OM314828	OM222960	OL417407
8	W-12	*Ae. columnaris*		189839	*Aegilops*	AZE	MW447528	OM314829	OM222961	OL417408
9	W-14	*Ae. comosa*	CYP, TUR, BGR, GRC	K024003	*Comopyrum*	-	MW447529	OM314830	OM222962	OL417409
10	W-15	*Ae. comosa*		K024002	*Comopyrum*	-	MW447530	OM314831	OM222963	OL417410
11	W-17	*Ae. comosa*		K024000	*Comopyrum*	-	MW447531	OM314832	OM222964	OL417411
12	W-18	*Ae. comosa*		K023999	*Comopyrum*	-	MW447532	OM314833	OM222965	OL417412
13	W-19	*Ae. comosa*		K023998	*Comopyrum*	-	MW447533	OM314834	OM222966	OL417413
14	W-20	*Ae. comosa*		801749	*Comopyrum*	CHE	MW447534	OM314835	OM222967	OL417414
15	W-22	*Ae. comosa*		208974	*Comopyrum*	-	MW447535	OM314836	OM222968	OL417415
16	W-23	*Ae. comosa*		206900	*Comopyrum*	TUR	MW447536	OM314837	OM222969	OL417416
17	W-24	*Ae. comosa*		206898	*Comopyrum*	TUR	MW447537	OM314838	OM222970	OL417417
18	W-27	*Ae. crassa*	AFG, IRN, IRQ, JOR, LBN, SYR, TUR, ARM, KAZ, KGZ, TJK, TKM, UZB	801574	*Vertebrata*	RUS	MW447538	OM314839	OM222971	OL417418
19	W-28	*Ae. crassa*		801599	*Vertebrata*	RUS	MW447539	OM314840	OM222972	OL417419
20	W-29	*Ae. crassa*		801598	*Vertebrata*	RUS	MW447540	OM314841	OM222973	OL417420
21	W-30	*Ae. crassa*		208983	*Vertebrata*	UZB	MW447541	OM314842	OM222974	OL417421

*Source: USDA, Agricultural Research Service, National Plant Germplasm System. (2022). Germplasm Resources Information Network (GRIN Taxonomy). http://npgsweb.ars-grin.gov/gringlobal/taxon/taxonomydetail?id=100015. [Accessed September 7 2022].

The country code (ISO 3166-1 alpha-3 code)

Supplementary Tables 1. Detailed descriptions of 84 *Aegilops* accessions. (2/4) (Continued)

No.	Sample No.	Taxon	Geographical distribution	Acc. No.	Section	Origin	ITS2	*mat*K	*rbc*L	*psb*M-*pet*N
22	W-31	*Ae. crassa*		208982	*Vertebrata*	KAZ	MW447542	OM314843	OM222975	OL417422
23	W-32	*Ae. crassa*		206909	*Vertebrata*	RUS	MW447543	OM314844	OM222976	OL417423
24	W-33	*Ae. crassa*		189843	*Vertebrata*	UZB	MW447544	OM314845	OM222977	OL417424
25	W-34	*Ae. cylindrica*	AFG, IRN, IRQ, ISR, JOR, LBN, TUR, RUS, ARM, AZE, GEO, RUS, KAZ, KGZ, TJK, TKM, UZB, PAK, HUN, SVK, MDA, RUS, UKR, BGR, GRC, HRV, MKD, ROU, SRB, SVN	801618	*Cylindropyrum*	RUS	MW447545	OM314846	OM222978	OL417425
26	W-35	*Ae. cylindrica*		801617	*Cylindropyrum*	RUS	MW447546	OM314847	OM222979	OL417426
27	W-36	*Ae. cylindrica*		801616	*Cylindropyrum*	RUS	MW447547	OM314848	OM222980	OL417427
28	W-37	*Ae. cylindrica*		204621	*Cylindropyrum*	-	MW447548	OM314849	OM222981	OL417428
29	W-38	*Ae. cylindrica*		189845	*Cylindropyrum*	UKR	MW447549	OM314850	OM222982	OL417429
30	W-39	*Ae. cylindrica*		189844	*Cylindropyrum*	TKM	MW447550	OM314851	OM222983	OL417430
31	W-40	*Ae. cylindrica*		158190	*Cylindropyrum*	-	MW447551	OM314852	OM222984	OL417431
32	W-5	*Ae. geniculata*	ESP, DZA, EGY, LBY, MAR, TUN, CYP, IRQ, ISR, JOR, LBN, SYR, TUR, AZE, GEO, CHE, HUN, UKR, ALB, BGR, BIH, GRC, HRV, ITA, MKD, MLT, MNE, ROU, SRB, SVN, ESP, FRA, PRT, USA	208979	*Aegilops*	LBN	MW447552	OM314853	OM222985	OL417432
33	W-41	*Ae. geniculata*		K024018	*Aegilops*	-	MW447553	OM314854	OM222986	OL417433
34	W-42	*Ae. geniculata*		231189	*Aegilops*	ROM	MW447554	OM314855	OM222987	OL417434
35	W-110	*Ae. geniculata*		208975	*Aegilops*	AZE	MW447555	OM314856	OM222988	OL417435
36	W-43	*Ae. juvenalis*		K024021	*Vertebrata*	-	MW447556	OM314857	OM222989	OL417436
37	W-45	*Ae. juvenalis*	IRQ, SYR, AZE	204630	*Vertebrata*	-	MW447557	OM314858	OM222990	OL417437
38	W-46	*Ae. juvenalis*		158208	*Vertebrata*	-	MW447558	OM314859	OM222991	OL417438
39	W-50	*Ae. longissima*		801576	*Sitopsis*	RUS	MW447559	OM314860	OM222992	OL417439
40	W-51	*Ae. longissima*	EGY, ISR, JOR	800260	*Sitopsis*	JOR	MW447560	OM314861	OM222993	OL417440
41	W-52	*Ae. longissima*		206890	*Sitopsis*	ISR	MW447561	OM314862	OM222994	OL417441
42	W-61	*Ae. neglecta*		206896	*Aegilops*	ITA	MW447562	OM314863	OM222995	OL417442

*Source: USDA, Agricultural Research Service, National Plant Germplasm System. (2022). Germplasm Resources Information Network (GRIN Taxonomy). http://npgsweb.ars-grin.gov/gringlobal/taxon/taxonomydetail?id=100015. [Accessed September 7 2022].

The country code (ISO 3166-1 alpha-3 code)

Supplementary Tables 1. Detailed descriptions of 84 *Aegilops* accessions. (3/4) (Continued)

No.	Sample No.	Taxon	Geographical distribution	Acc. No.	Section	Origin	ITS2	*mat*K	*rbc*L	*psb*M-*pet*N
43	W-62	*Ae. neglecta*	DZA, MAR, TUN, IRN,	206895	*Aegilops*	TUR	MW447563	OM314864	OM222996	OL417443
44	W-63	*Ae. neglecta*	IRQ, ISR, SYR, TUR	189838	*Aegilops*	AZE	MW447564	OM314865	OM222997	OL417444
45	W-70	*Ae. searsii*	ISR, JOR, LBN, SYR	800259	*Sitopsis*	SYR	MW447565	OM314866	OM222998	OL417445
46	W-71	*Ae. searsii*		900131	*Sitopsis*	-	MW447566	OM314867	OM222999	OL417446
47	W-72	*Ae. searsii*		206891	*Sitopsis*	ISR	MW447567	OM314868	OM223000	OL417447
48	W-74	*Ae. sharonensis*		801581	*Sitopsis*	RUS	MW447568	OM314869	OM223001	OL417448
49	W-75	*Ae. sharonensis*	ISR, LBN	206892	*Sitopsis*	-	MW447569	OM314870	OM223002	OL417449
50	W-82	*Ae. sharonensis*		900130	*Sitopsis*	-	MW447570	OM314871	OM223003	OL417450
51	W-81	*Ae. speltoides*	IRN, IRQ, ISR, LBN,	801583	*Sitopsis*	RUS	MW447571	OM314872	OM223004	OL417451
52	W-83	*Ae. speltoides*	SYR, TUR, BGR, GRC	204625	*Sitopsis*	-	MW447572	OM314873	OM223005	OL417452
53	W-90	*Ae. tauschii*		302677	*Vertebrata*	TUR	MW447573	OM314874	OM223006	OL417453
54	W-91	*Ae. tauschii*		269551	*Vertebrata*	TUR	MW447574	OM314875	OM223007	OL417454
55	W-92	*Ae. tauschii*	AFG, IRN, IRQ, SYR,	269550	*Vertebrata*	TUR	MW447575	OM314876	OM223008	OL417455
56	W-93	*Ae. tauschii*	TUR, RUS, ARM, AZE,	269549	*Vertebrata*	TUR	MW447576	OM314877	OM223009	OL417456
57	W-94	*Ae. tauschii*	GEO, KAZ, KGZ, TJK,	269555	*Vertebrata*	AFG	MW447577	OM314878	OM223010	OL417457
58	W-95	*Ae. tauschii*	TKM, UZB, CHN, IND,	302676	*Vertebrata*	CHN	MW447578	OM314879	OM223011	OL417458
59	W-96	*Ae. tauschii*	PAK, UKR, USA	302675	*Vertebrata*	CHN	MW447579	OM314880	OM223012	OL417459
60	W-104	*Ae. tauschii*		900129	*Vertebrata*	-	MW447580	OM314881	OM223013	OL417460
61	W-105	*Ae. tauschii*		K024006	*Vertebrata*	-	MW447581	OM314882	OM223014	OL417461
62	W-97	*Ae. triuncialis*		801614	*Aegilops*	RUS	MW447582	OM314883	OM223015	OL417462
63	W-98	*Ae. triuncialis*		801613	*Aegilops*	RUS	MW447583	OM314884	OM223016	OL417463

*Source: USDA, Agricultural Research Service, National Plant Germplasm System. (2022). Germplasm Resources Information Network (GRIN Taxonomy). http://npgsweb.ars-grin.gov/gringlobal/taxon/taxonomydetail?id=100015. [Accessed September 7 2022].

The country code (ISO 3166-1 alpha-3 code)

Supplementary Tables 1. Detailed descriptions of 84 *Aegilops* accessions. (4/4) (Continued)

**No.**	**Sample No.**	**Taxon**	**Geographical distribution**	**Acc. No.**	**Section**	**Origin**	**ITS2**	***mat*K**	***rbc*L**	***psb*M-*pet*N**
64	W-99	*Ae. triuncialis*	DZA, MAR, KWT, AFG, CYP, IRN,	801612	*Aegilops*	RUS	MW447584	OM314885	OM223017	OL417464
65	W-100	*Ae. triuncialis*	IRQ, ISR, LBN, SYR, TUR, ARM,	801611	*Aegilops*	RUS	MW447585	OM314886	OM223018	OL417465
66	W-101	*Ae. triuncialis*	AZE, GEO, RUS, KAZ, KGZ, TJK,	208985	*Aegilops*	CHE	MW447586	OM314887	OM223019	OL417466
67	W-102	*Ae. triuncialis*	TKM, UZB, PAK, UKR, ALB,	208984	*Aegilops*	ESP	MW447587	OM314888	OM223020	OL417467
68	W-103	*Ae. triuncialis*	BGR, GRC, ITA, MKD, SVN, ESP, FRA, PRT	204619	*Aegilops*	-	MW447588	OM314889	OM223021	OL417468
69	W-9	*Ae. umbellulata*		208981	*Aegilops*	IRN	MW447589	OM314890	OM223022	OL417469
70	W-106	*Ae. umbellulata*		801591	*Aegilops*	RUS	MW447590	OM314891	OM223023	OL417470
71	W-108	*Ae. umbellulata*	IRN, IRQ, LBN, SYR,	801579	*Aegilops*	RUS	MW447591	OM314892	OM223024	OL417471
72	W-109	*Ae. umbellulata*	TUR, ARM, AZE, GRC	208976	*Aegilops*	TUR	MW447592	OM314893	OM223025	OL417472
73	W-111	*Ae. umbellulata*		189840	*Aegilops*	AZE	MW447593	OM314894	OM223026	OL417473
74	W-112	*Ae. umbellulata*		158191	*Aegilops*	TUR	MW447594	OM314895	OM223027	OL417474
75	W-113	*Ae. uniaristata*		K024007	*Comopyrum*	-	MW447595	OM314896	OM223028	OL417475
76	W-114	*Ae. uniaristata*	TUR, ALB, GRC, HRV, ITA, MKD	302223	*Comopyrum*	GRC	MW447596	OM314897	OM223029	OL417476
77	W-115	*Ae. uniaristata*		208977	*Comopyrum*	-	MW447597	OM314898	OM223030	OL417477
78	W-116	*Ae. uniaristata*		206903	*Comopyrum*	TUR	MW447598	OM314899	OM223031	OL417478
79	W-118	*Ae. ventricosa*		801573	*Vertebrata*	RUS	MW447599	OM314900	OM223032	OL417479
80	W-119	*Ae. ventricosa*		801572	*Vertebrata*	RUS	MW447600	OM314901	OM223033	OL417480
81	W-120	*Ae. ventricosa*	DZA, EGY, LBY, MAR,	801571	*Vertebrata*	RUS	MW447601	OM314902	OM223034	OL417481
82	W-121	*Ae. ventricosa*	TUN, HRV, ITA, ESP, FRA	206913	*Vertebrata*	-	MW447602	OM314903	OM223035	OL417482
83	W-122	*Ae. ventricosa*		206912	*Vertebrata*	-	MW447603	OM314904	OM223036	OL417483
84	W-123	*Ae. ventricosa*		204629	*Vertebrata*	-	MW447604	OM314905	OM223037	OL417484

*Source: USDA, Agricultural Research Service, National Plant Germplasm System. (2022). Germplasm Resources Information Network (GRIN Taxonomy). http://npgsweb.ars-grin.gov/gringlobal/taxon/taxonomydetail?id=100015. [Accessed September 7 2022].

The country code (ISO 3166-1 alpha-3 code)

Supplementary Tables 2. The best-fit replacement model used to construct the maximum likelihood tree for candidate DNA barcode.

**Regions**	**Best-fit replacement model**
I	K2P
M	TN + F + I + G4
R	K2P + I
P	F81+ F
I + M	TN + F + I + G4
I + R	TIM3e + I + G4
I + P	TPM2u + F + G4
M + R	TN + F + I + G4
M + P	TIM3 + F + I + G4
R + P	TN + F + I
I + M + R	TIM3 + F + I + G4
I + M + P	TN + F + I + G4
I + R + P	TIM3 + F + I + G4
M + R + P	TN + F + I + G4
I + M + R + P	TN + F + I + G4

Supplementary Tables 3. Number of intraspecific variable characters per species in ITS2, *mat*K, *rbc*L, *psb*M-*pet*N.

Species	Intraspecific variations			
	ITS2	*mat*K	*rbc*L	*psb*M-*pet*N
Aegilops biuncialis	0	2	2	2
Aegilops columnaris	0	1	1	0
Aegilops comosa	0	9	2	7
Aegilops crassa	1	8	1	0
Aegilops cylindrica	0	5	2	0
Aegilops geniculata	0	6	2	0
Aegilops juvenalis	0	2	1	0
Aegilops longissima	0	5	1	1
Aegilops neglecta	0	4	1	0
Aegilops searsii	0	4	2	0
Aegilops sharonensis	0	1	2	0
Aegilops speltoides	0	1	1	1
Aegilops tauschii	0	9	2	1
Aegilops triuncialis	0	9	3	0
Aegilops umbellulata	0	9	4	2
Aegilops uniaristata	0	6	1	0
Aegilops ventricosa	0	13	3	0
All 17 species	26	28	6	10

Supplementary Tables 4. The Best match, Best close match and All species barcodes analysis comparing the identification ability of 15 candidate barcodes. Correct, both sequences were from the same species. Incorrect, Any mismatch. Ambiguous, Several cases with equally good best matches from different species.

Regions	Best match, n (%)			Best close match, n (%)				All species barcode			
	**Correct**	**Ambiguous**	**Incorrect**	**Correct**	**Ambiguous**	**Incorrect**	**No match**	**Correct**	**Ambiguous**	**Incorrect**	**No match**
I	49 (56.97%)	35 (40.69%)	2 (2.32%)	49 (56.97%)	35 (40.69%)	2 (2.32%)	0 (0.0%)	82 (95.34%)	4 (4.65%)	0 (0.0%)	0 (0.0%)
M	32 (37.2%)	46 (53.48%)	8 (9.3%)	32 (37.2%)	46 (53.48%)	8 (9.3%)	0 (0.0%)	14 (16.27%)	72 (83.72%)	0 (0.0%)	0 (0.0%)
R	5 (5.81%)	79 (91.86%)	2 (2.32%)	5 (5.81%)	79 (91.86%)	2 (2.32%)	0 (0.0%)	1 (1.16%)	85 (98.83%)	0 (0.0%)	0 (0.0%)
P	15 (17.44%)	70 (81.39%)	1 (1.16%)	15 (17.44%)	69 (80.23%)	1 (1.16%)	1 (1.16%)	49 (56.97%)	36 (41.86%)	0 (0.0%)	1 (1.16%)
I + M	60 (69.76%)	20 (23.25%)	6 (6.97%)	60 (69.76%)	20 (23.25%)	6 (6.97%)	0 (0.0%)	40 (46.51%)	46 (53.48%)	0 (0.0%)	0 (0.0%)
I + R	60 (69.76%)	23 (26.74%)	3 (3.48%)	60 (69.76%)	23 (26.74%)	3 (3.48%)	0 (0.0%)	34 (39.53%)	52 (60.46%)	0 (0.0%)	0 (0.0%)
I + P	70 (81.39%)	14 (16.27%)	2 (2.32%)	70 (81.39%)	14 (16.27%)	0 (0.0%)	2 (2.32%)	60 (69.76%)	24 (27.9%)	0 (0.0%)	2 (2.32%)
M + R	32 (37.2%)	44 (51.16%)	10 (11.62%)	32 (37.2%)	44 (51.16%)	10 (11.62%)	0 (0.0%)	11 (12.79%)	75 (87.2%)	0 (0.0%)	0 (0.0%)
M + P	49 (56.97%)	26 (30.23%)	11 (12.79%)	49 (56.97%)	26 (30.23%)	10 (11.62%)	1 (1.16%)	16 (18.6%)	69 (80.23%)	0 (0.0%)	1 (1.16%)
R + P	27 (31.39%)	50 (58.13%)	9 (10.46%)	27 (31.39%)	50 (58.13%)	8 (9.3%)	1 (1.16%)	10 (11.62%)	75 (87.2%)	0 (0.0%)	1 (1.16%)
I + M + R	62 (72.09%)	13 (15.11%)	11 (12.79%)	62 (72.09%)	13 (15.11%)	11 (12.79%)	0 (0.0%)	37 (43.02%)	49 (56.97%)	0 (0.0%)	0 (0.0%)
I + M + P	73 (84.88%)	9 (10.46%)	4 (4.65%)	73 (84.88%)	9 (10.46%)	2 (2.32%)	2 (2.32%)	51 (59.3%)	33 (38.37%)	0 (0.0%)	2 (2.32%)
I + R + P	70 (81.39%)	11 (12.79%)	5 (5.81%)	70 (81.39%)	11 (12.79%)	3 (3.48%)	2 (2.32%)	53 (61.62%)	31 (36.04%)	0 (0.0%)	2 (2.32%)
M + R + P	51 (59.3%)	20 (23.25%)	15 (17.44%)	51 (59.3%)	20 (23.25%)	14 (16.27%)	1 (1.16%)	13 (15.11%)	72 (83.72%)	0 (0.0%)	1 (1.16%)
I + M + R + P	76 (88.37%)	4 (4.65%)	6 (6.97%)	76 (88.37%)	4 (4.65%)	4 (4.65%)	2 (2.32%)	50 (58.13%)	34 (39.53%)	0 (0.0%)	2 (2.32%)

I, ITS2; M, *mat*K; R, *rbc*L; P, *psb*M-*pet*N.

Supplementary Tables 5. Analyze the identification efficiency of candidate DNA barcodes based on the maximum likelihood (ML) tree results.

DNA barcode	Classified species	Number of species classified
I	*Ae. searsii, Ae. comosa, Ae. crassa, Ae. tauschii, Ae. speltoides, Ae. uniaristata, Ae. ventricosa, Ae. columnaris, Ae. neglecta, Ae. sharonensis*	10
M	*Ae. longissima, Ae. searsii, Ae. triuncialis, Ae. cylindrica, Ae. tauschii*	5
R	*Ae. speltoides*	1
P	*Ae. geniculata, Ae. uniaristata, Ae. speltoides*	3
I+M	*Ae. longissima, Ae. triuncialis, Ae. crassa, Ae. uniaristata, Ae. ventricosa, Ae. sharonensis, Ae. columnaris, Ae. cylindrica, Ae. tauschii, Ae. speltoides*	10
I+R	*Ae. triuncialis, Ae. speltoides, Ae. tauschii, Ae. uniaristata, Ae. crassa, Ae. columnaris, Ae. sharonensis*	7
I+P	*Ae. searsii, Ae. sharonensis, Ae. columnaris, Ae. neglecta, Ae. geniculata, Ae. triuncialis, Ae. cylindrica, Ae. tauschii, Ae. ventricosa, Ae. crassa, Ae. speltoides, Ae. uniaristata*	12
M+R	*Ae. triuncialis, Ae. cylindrica, Ae. tauschii, Ae. speltoides, Ae. searsii, Ae. longissima*	6
M+P	*Ae. searsii, Ae. longissima, Ae. triuncialis, Ae. speltoides*	4
R+P	*Ae. speltoides, Ae. geniculata, Ae. uniaristata*	3
I+M+R	*Ae. columnaris, Ae. uniaristata, Ae. ventricosa, Ae. crassa, Ae. sharonensis, Ae. longissima, Ae. searsii, Ae. triuncialis, Ae. speltoides, Ae. cylindrica, Ae. tauschii*	11
I+M+P	*Ae. longissima, Ae. columnaris, Ae. sharonensis, Ae. uniaristata, Ae. triuncialis, Ae. crassa, Ae. speltoides, Ae. cylindrica, Ae. tauschii, Ae. ventricosa, Ae. geniculata*	11
I+R+P	*Ae. columnaris, Ae. sharonensis, Ae. geniculata, Ae. speltoides, Ae. crassa, Ae. triuncialis, Ae. cylindrica, Ae. tauschii, Ae. ventricosa, Ae. uniaristata*	10
M+R+P	*Ae. cylindrica, Ae. tauschii, Ae. searsii, Ae. triuncialis, Ae. longissima*	5
I+M+R+P	*Ae. uniaristata, Ae. cylindrica, Ae. tauschii, Ae. ventricosa, Ae. crassa, Ae. speltoides, Ae. neglecta, Ae. sharonensis, Ae. geniculata, Ae. triuncialis, Ae. longissima, Ae. searsii, Ae. columnaris*	13

I, ITS2; M, *mat*K; R, *rbc*L; P, *psb*M-*pet*N.
